# Thermal environment and indices: an analysis for effectiveness in operational weather applications in a Mediterranean city (Athens, Greece)

**DOI:** 10.1007/s00484-023-02572-7

**Published:** 2023-11-15

**Authors:** Katerina Pantavou, Vassiliki Kotroni, Konstantinos Lagouvardos

**Affiliations:** Institute for Environmental Research and Sustainable Development, National Observatory of Athens, 15236 Penteli, Athens Greece

**Keywords:** Thermal index, Thermal conditions, PET, UTCI, NET, Operational weather application

## Abstract

The large number of thermal indices introduced in the literature poses a challenge to identify the appropriate one for a given application. The aim of this study was to examine the effectiveness of widely used indices in quantifying the thermal environment for operational weather applications within a Mediterranean climate. Eight indices (six simple and two thermo-physiological) were considered, i.e., apparent temperature, heat index, humidex, net effective temperature (NET), physiologically equivalent temperature (PET), universal thermal climate index (UTCI), wet-bulb globe temperature, and wind chill temperature. They were estimated using hourly meteorological data between 2010 and 2021, recorded in 15 stations from the Automatic Weather Station Network of the National Observatory of Athens in the Athens metropolitan area, Greece. The statistical analysis focused on examining indices’ sensitivity to variations of the thermal environment. NET, PET, and UTCI were evaluated as suitable for operational use, assessing both cool and warm environments, and extending their estimations to the entire range of their assessment scales. NET and PET often tended to classify thermal perception in the negative categories of their scales, with 63% of NET and 56% of PET estimations falling within the range of cool/slightly cool to very cold. UTCI estimations in the negative categories accounted for 25.8% (*p* < 0.001), while most estimations were classified in the neutral category (53.1%). The common occasions of extreme warm conditions in terms of both air temperature (Tair) and NET was 77.7%, Tair and UTCI 64.4%, and Tair and PET 33.6% (*p* < 0.001). According to the indices considered and the method followed, NET and UTCI satisfied sufficiently the requirements for operational use in the climate conditions of the Mediterranean climate.

**Supplementary Information:**

The online version contains supplementary material available at 10.1007/s00484-023-02572-7.

## Introduction

Human biometeorology is an interdisciplinary science that studies the interactions between atmospheric processes and humans focusing greatly on the impact of the thermal environment on health and well-being (ISB 2021). Aspects and applications explored include architecture (urban planning, designing open spaces, recreation areas and buildings, and building materials), energy efficiency and conservation, tourism industry, work performance and productivity, occupational and public health, weather forecasting and warning systems, and research related to climate change (Fischereit and Schlünzen 2018; Flouris et al. 2018; Di Napoli et al. 2021; Katavoutas et al. 2021; Romaszko et al. 2022; Tseliou et al. 2022). Popular measures for the assessment of thermal environment are the thermal indices (Coccolo et al. 2016; de Freitas and Grigorieva 2017).

A large number of thermal indices have been introduced in the literature over the years (de Freitas and Grigorieva 2015, 2017; Coccolo et al. 2016; Potchter et al. 2018). They were developed considering different rational approaches, incorporated variables, applications, and type of outputs (i.e., thermal sensation, comfort, stress) (de Freitas and Grigorieva 2015). Some are suitable to warm environmental conditions such as heat index (HI) (Steadman 1979; Rothfusz 1990), humidex (HU) (Masterson and Richardson 1979), and wet-bulb globe temperature (WBGT) (Yaglou and Minard 1957; BOM 2010), while others are suitable to cool environments such as wind chill temperature (WCT) (ASHRAE 1997). Indices evaluating both cool and warm environments were also developed such as apparent temperature (AT) (Steadman 1979; BOM 2010), net effective temperature (NET) (Li and Chan 2000), and universal indices such as physiologically equivalent temperature (PET) (Mayer and Höppe 1987; Höppe 1999) and universal thermal climate index (UTCI) (Jendritzky et al. 2012). The most easily calculated thermal indices consider only meteorological variables (i.e., AT, HI, HU, NET, WBGT, WCT). The more complex thermo-physiological indices are based on human energy balance model and consider additionally radiation fluxes and personal factors such as clothing insulation and activity level (i.e., PET and UTCI).

This variety of indices raises considerations over the use of the most appropriate one in an application under specified conditions and within a selected microclimate. Studies have examined various characteristics of the indices such as: their applicability (i.e., relevance and suitability for assessing the subjective thermal perception), their accuracy (i.e., alignment with the subjective thermal perception), their practicability (i.e., feasibility and usability with which an index can be practically applied considering factors such as data, resources, and interpretation), and their appropriateness (i.e., overall relevance and suitability for accurately assessing the thermal environment). Based on the applicability and the accuracy of the thermal indices, indices’ thresholds have been redefined for different climatic zones (Potchter et al. 2018). Several of the studies have focused on the Mediterranean climate (Cohen et al. 2013; Pantavou et al. 2014, 2020; Salata et al. 2016) and in particular the climatic zone of Athens, Greece (Pantavou et al. 2013, 2014; Pantavou and Lykoudis 2014). Arguments support that indices’ accuracy could be less important than their applicability and practicability while indices’ appropriateness depends on the aim of the study and the application methodology (Epstein and Moran 2006; Matzarakis 2021).

Nowadays, thermo-physiological indices are considered more appropriate for the assessment of thermal environment (Matzarakis 2021). The indices identified as the most common in the studies of outdoor thermal perception are PET, predicted mean vote (PMV), standard effective temperature (SET*), and UTCI (Potchter et al. 2018). Simple approaches such as AT, HI, HU, NET, WBGT, and WCT are used operationally in applications of international weather agencies (HNMS 2023; Li and Chan 2000; BOM 2010; Goverment of Canada 2021; Hong Kong Observatory 2022; NOAA 2022; The Cyprus Institute 2022; Met Office 2023) due to the easily accessible data enabled in their computation avoiding estimations of radiation fluxes. Recently, UTCI has been incorporated in the forecasting procedure at several weather institutes and is currently in use at four European countries (i.e., Italy, Portugal, Poland, Czech) (Di Napoli et al. 2021) while it is also implemented at the European Centre for Medium-Range Forecasts (ECMWF). Some efforts have been made for the estimation of PET operationally (Giannaros et al. 2018); however, at least to our knowledge, there is no operational use of PET at the present.

The aim of this study was to examine the effectiveness of thermal indices widely used in research (i.e., PET, UTCI) and weather agencies (i.e., AT, HI, HU, NET, WBGT, and WCT) for the assessment of thermal environment in operational weather applications in the Mediterranean climate. Τhe effectiveness refers to the ability of thermal indices to provide meaningful and relevant information, to capture variations in temperature and related factors, to be popular and practical, and to aid decision-making process.

## Materials and methods

### Thermal indices

Eight thermal indices were considered in this study, AT, HI, HU, NET, PET, UTCI, WBGT, and WCT (Table 1, Online Resource Tables S1). They were identified as those widely used either for research or operational purposes in international weather and climate agencies and organizations. All indices examined provide an output of thermal dimension (°C) which can be assigned to a class of an assessment scale expressing the degree of human thermal perception, i.e., comfort, sensation, or stress (Online Resource Tables S2 and S3). For the analysis and in order to facilitate the comparison between indices’ estimations and the interpretation of results, the estimated degree of thermal perception was assigned to a common for all indices numerical scale, namely, index level (range between − 6 and 5; Online Resource Tables S2 and S3). As the indices’ scales pertain to the assessment of diverse characteristics such as comfort, sensation, and stress, the unified scale employed in the present study delineates the category level in each assessment scale. Zero level (0) denotes the indifference category (e.g., neutral, no discomfort, comfortable, no danger, no thermal stress). Positive (negative) levels from 1 to 5 (− 1 to − 6) denote increasing intensity of the warm (cool) categories of the indices assessment scale.

**Table 1:** Summary of thermal indices features considered in the present study

Index	Abbreviation	Unit	Source	Conditions applied	Variables
Apparent temperature	AT_warm_	°C	(Steadman 1984)	Warm	Tair, Rh, WS
Heat index	HI	°C	(Rothfusz 1990)	Warm	Tair, Rh
Humidex	HU	°C	(Masterson and Richardson 1979)	Warm	Tair, Rh
Net effective temperature	NET	°C	(Li and Chan 2000)	All	Tair, Rh, WS
Physiologically equivalent temperature	PET	°C	(Höppe 1984, 1999; Mayer and Höppe 1987)	All	Tair, Rh, WS, SR
Universal thermal climate index	UTCI	°C	(Fiala et al. 2001; Bröde et al. 2012)	All	Tair, Rh, WS, SR
Wet-bulb globe temperature	WBGT	°C	(Yaglou and Minard 1957)	Warm	Tair, Rh
Wind chill temperature	WCT	°C	(ASHRAE 1997)	Cool	Tair, Rh, WS

AT, HI, HU, NET, WBGT, and WCT are simple indices estimated using a simple algebraic formula (Online Resource Tables S1) and standard meteorological variables: air temperature (Tair, °C), relative humidity (Rh, %), and wind speed (WS, m/s). These indices are used operationally in international weather agencies, e.g., in Australia, Canada, China, Cyprus, Greece, the UK, and the USA (HNMS 2023; Li and Chan 2000; BOM 2010; Goverment of Canada 2021; Hong Kong Observatory 2022; NOAA 2022; The Cyprus Institute 2022; Met Office 2023). In this study, AT was calculated for Tair ≥ 20 °C (namely, AT_warm_) in order to adopt the assessment scale focused on warm conditions (Brimicombe et al. 2022).

The PET and the UTCI are thermo-physiological indices based on multi-node human heat balance models and consider radiation fluxes for the estimation of human thermal response. They can be estimated using Tair, Rh, WS, and global solar radiation (SR, W/m^2^) (Matzarakis et al. 2007, 2010). PET and UTCI are suggested as the most commonly used thermal indices along with PMV and SET* for assessing thermal perception in the published literature (Potchter et al. 2018, 2022).

PMV and SET* were not considered in the analysis since they were not found to be used operationally at the present and they showed lower applicability in the Mediterranean climate compared to PET and UTCI (Pantavou et al. 2013).

### Data and estimation of thermal indices

**Fig. 1:**
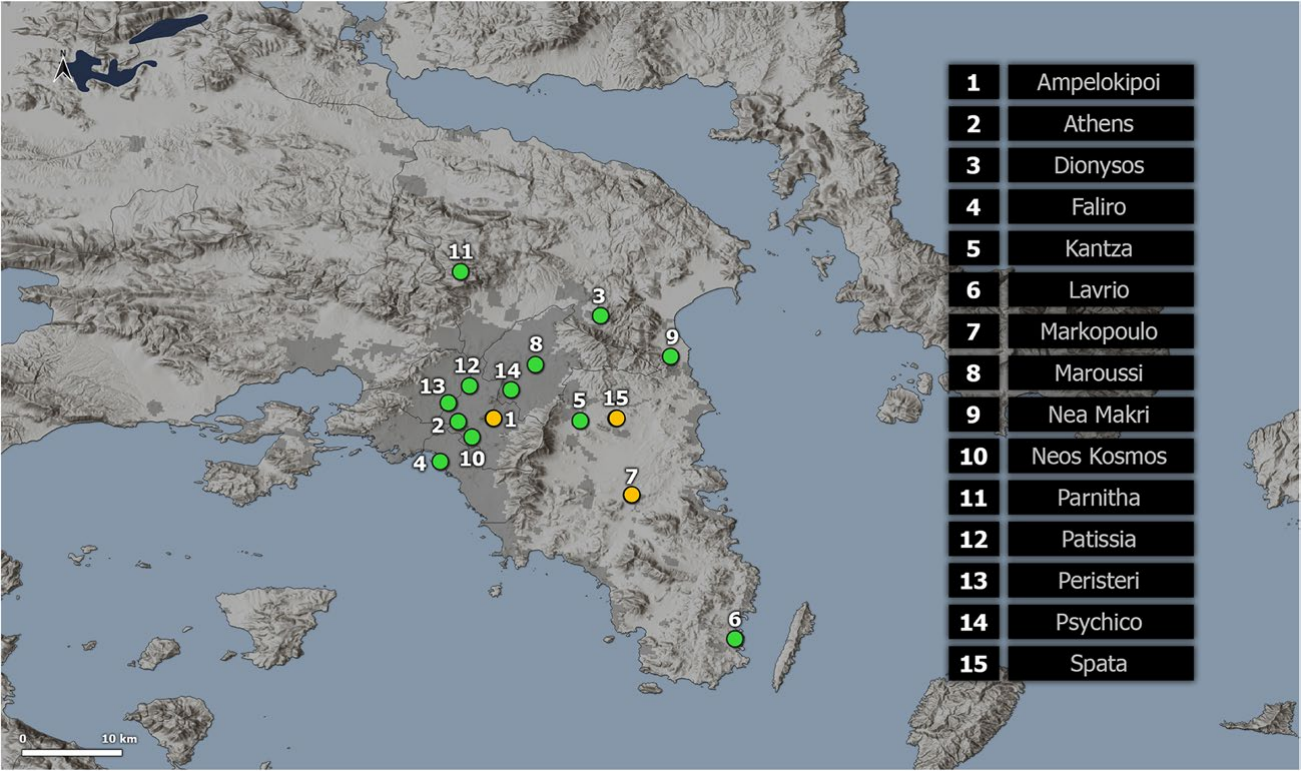
Stations in the Athens metropolitan area, Greece, operated by the METEO unit of the National Observatory of Athens, which were included in this study (stations with solar radiation sensors are in yellow)

Hourly data of Tair, Rh, and WS for the period 2010–2021 were derived from 15 surface weather stations in Athens metropolitan area, Greece (Fig. 1, Online Resource Tables S4) operated by the METEO Unit at the National Observatory of Athens (NOA) (Lagouvardos et al. 2017). Global solar radiation data were available in 3 out of the 15 stations of the network (Online Resource Table S4). Thus, hourly SR data for those stations with no SR sensor were derived from the closest station that measures global solar radiation. The maximum distance considered was 33 km between Markopoulo and Lavrio stations.

A Python programming language script was developed for the estimation of the thermal indices. PET was estimated using the package for thermal comfort research (Tartarini and Schiavon 2020) and setting clothing insulation to 0.9 clo (0, no clothing; 1, business suite) and the activity level to 1.37 met (standing person).

### Data analysis

The statistical analysis focused on examining indices’ sensitivity to variations of the thermal environment. Statistical measures including mean, standard deviation, maximum and minimum values, and 1%, 5%, 10%, 25%, 50%, 75% 90%, 95%, and 99% percentiles (*p*) were used to describe thermal perception according to indices’ estimations. The frequency of the estimated levels was examined as well. This analysis shows possible tendency of classifying thermal conditions in certain categories of the assessment scales. The two-sample test of proportions was used to test the equality of proportions. The one-way analysis of variance (ANOVA) was used to determine whether the mean Tair differs among the categories of the assessment scales and the *t*-test to determine the equality of Tair means between the indices. Pearson’s correlation was used as a measure of association between Tair and the indices. A *p* value less than 0.05 indicates statistical significance.

Moreover, the indices’ ability to reproduce extreme thermal conditions was examined. It was explored whether extreme thermal conditions defined by Tair (i.e., the most common measure of cool or warm weather) are in accordance with those defined by the indices. Thresholds for extreme thermal conditions were set equal to the 95% of the daily maximum and the 5% of the daily minimum Tair (Di Napoli et al. 2019; Founda et al. 2022). The same approach was used for the thermal indices. The thresholds of 5% and 95% instead of 1% and 99%, respectively, were employed to achieve a broad range of extreme thermal conditions. The occurrences of extreme thermal conditions were defined as the exceedances of the daily maximum Tair/thermal index from the respective 95% or 5% threshold in each station independently.

## Results

Table 2 presents the summary statistics of the overall measured meteorological variables and thermal indices’ categories for all 15 weather stations. The Tair ranged between − 12 and 43.4 °C. The estimated thermal perception extended to the whole range of indices’ assessment scales except for AT and WCT (Table 2). The maximum estimated level of AΤ was 3, missing the level 4. WCT estimations were limited between − 2 and 0, missing levels above − 3 that could be justified by the fact that in Athens negative temperatures are quite rare (p1 is 0.9 °C, as denoted in Table 2).

**Table 2:** Summary statistics of hourly values of meteorological variables and thermal indices’ estimations and levels derived from 15 surface weather stations in the Athens metropolitan area, Greece

Variable	Index level	Mean	SD	Min	Max	p1	p5	p10	p25	p50	p75	p90	p95	p99
Tair (°C)		18.1	7.8	− 12	43.4	0.9	5.8	8.2	12.3	17.6	24.2	28.6	30.8	34.1
Rh (%)		65	17	10	100	28	36	42	52	65	77	86	91	98
WS (m/s)		2.1	2.2	0.5	16.7	0	0	0	1	1	2.7	5.0	6.7	10.8
SR (W/m^2^)		186	269	0	1292	0	0	0	0	5	332	659	788	896
AT_warm_ (°C)		26.0	4.5	7.2	48.4	16.5	19.1	20.4	22.7	25.8	29.2	32.1	33.8	36.7
HI (°C)		27.1	3.3	15.4	58.0	20.7	22.9	23.8	24.9	26.2	28.9	31.9	33.6	36.9
HU (°C)		30.1	4.9	16.0	56.8	20.8	22.7	23.8	26.3	29.8	33.6	36.8	38.6	41.6
NET (°C)		13.3	8.5	− 33.4	35.2	− 12.3	− 1.8	2.6	8.3	13.7	20.0	23.5	25.1	27.6
PET (°C)		16.1	11.5	− 21.0	57.6	− 6.8	− 1.0	2.0	7.5	15.4	23.7	32.5	36.7	42.7
UTCI (°C)		16.2	12.2	− 55.4	51.8	− 20.2	− 4.2	1.8	8.7	16.7	24.6	31.6	35.2	39.9
WBGT (°C)		25.5	3.0	16.4	42.4	19.7	21.0	21.7	23.2	25.3	27.6	29.6	30.7	32.5
WCT (°C)		5.5	4.2	− 15.2	11.4	− 7.7	− 3.0	− 3.8	3.2	6.5	8.6	9.9	10.4	11.1
AT_warm_	0 to 4	0.5	0.7	0	3	0	0	0	0	0	1	2	2	2
HI	0 to 4	0.5	0.7	0	4	0	0	0	0	0	1	1	2	2
HU	0 to 5	0.7	0.8	0	5	0	0	0	0	0	1	2	2	3
NET	− 3 to 3	− 0.6	1.4	− 3	3	− 3	− 3	− 2	− 2	− 1	0	2	2	3
PET	− 4 to 4	− 0.9	2.1	− 4	4	− 4	− 4	− 4	− 3	− 1	1	2	3	4
UTCI	− 5 to 4	0.0	1.1	− 5	4	− 3	− 2	− 1	− 1	0	0	1	2	3
WBGT	0 to 4	2.0	0.9	0	4	1	1	1	1	2	2	3	4	4
WCT	− 6 to 0	− 0.1	0.3	− 2	0	− 1	− 1	− 1	0	0	0	0	0	0

Abbreviations:* AT*, apparent temperature; *HI*, heat index; *HU*, humidex; *NET*, net effective temperature; *p*, percentile; *PET*, physiologically equivalent temperature; *Rh*, relative humidity; *Tair*, air temperature; *SD*, standard deviation; *SR*, global solar radiation; *UTCI*, Universal Thermal Climate Index; *WBGT*, wet-bulb globe temperature; *WCT*, wind chill temperature; WS, wind speed

AT_warm_ classified the 99% of its estimations up to level 2 (Table 2). On the other hand, WBGT tended to classify thermal perception to high levels of its assessment scale (i.e., the 90% percentile includes estimations in category 3 and the 95% in 4). The 99% percentile of categorical estimations of HU and UTCI was up to level 3, showing that HU and UTCI are sparing in classifying thermal perception to extreme warm categories of their assessment scales (i.e., level 5 for HU and 4 for UTCI). On the contrary, NET and PET reached their highest level at the 99% percentile (i.e., 3 for NET and 4 for PET). On the negative categories, NET and PET classified frequently perception to the extreme cool categories (i.e., level − 3 for NET and − 4 for PET) as 50% of the estimations was less or equal to level − 1. On the contrary, UTCI estimations showed a low intense mode of cool perception with 25% of its estimations classified below level − 1.

**Fig. 2:**
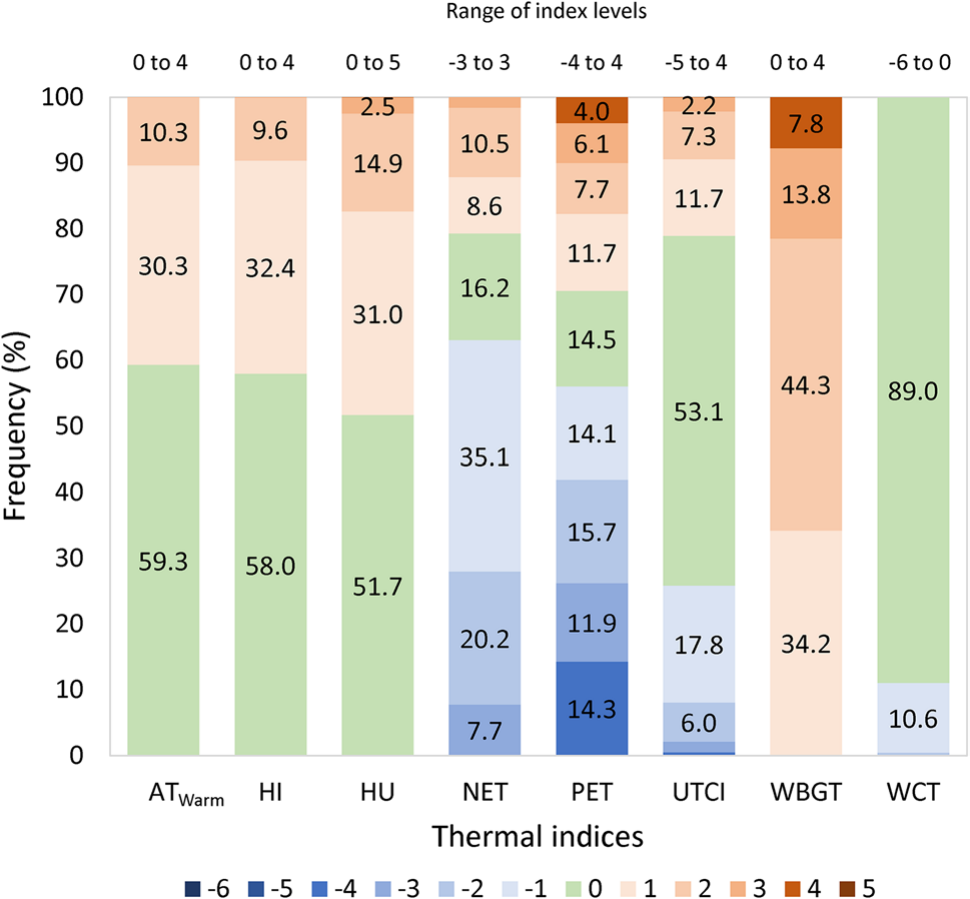
Distribution of indices’ estimations in the levels of their assessment scales. The indices were calculated using hourly data recorded in 15 surface weather stations in the Athens metropolitan area, Greece

The distribution of indices’ estimations (Fig. 2) showed that most estimations of UTCI was in level 0 (53.1%), NET in level − 1 (35.1%), and PET in − 2 (15.7%). Overall, the estimations classified in the negative levels of thermal perception were 63% for NET, 56% for PET, and 25.8% for UTCI (*p* ≤ 0.001). The frequency of estimations found in the positive levels were 20.7% for NET, 29.5% for PET, and 21.1% for UTCI (*p* ≤ 0.001; Fig. 2).

**Fig. 3:**
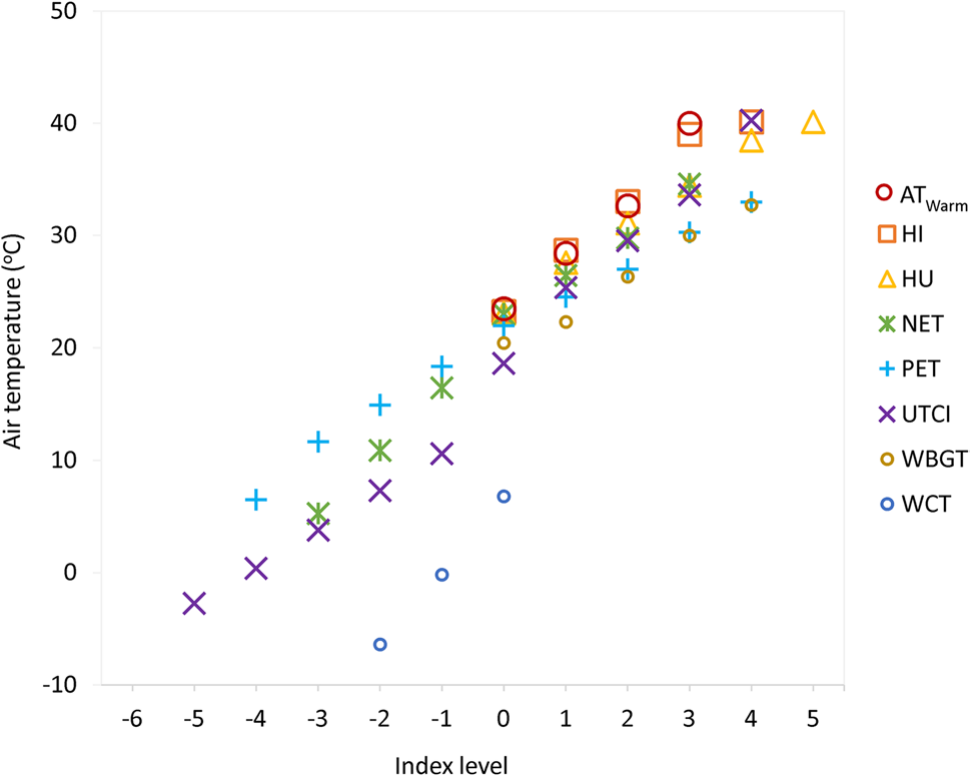
Mean air temperature for each level of thermal indices’ assessment scale

Analysis of variance showed statistically significant differences of Tair, Rh, WS, and SR means between the categories of the assessment scales for each index (*p* ≤ 0.001; Fig. 3 and Online Resource Figures S1-3). The mean Tair in level 0 was 18.6 °C for UTCI and 20.4 °C for WBGT, and above 23 °C for HI (23.2 °C), HU (23.1 °C), and NET (23 °C; *p* ≤ 0.001). The mean Rh was 65.6% for UTCI, 76.6% for WCT, and ranged between 55.8% and 59.5% for AT_warm_, HI, HU, NET, and PET (Online Resource Figures S1).

A statistically significant correlation was found between Tair and all indices (*p* ≤ 0.001). The correlation coefficients ranged between 0.88 (WBGT) and 0.96 (WCT). The coefficient was 0.94 for NET, 0.93 for PET, and 0.89 for UTCI.

### Extreme thermal conditions

Table 3 shows the thresholds of Tair and indices used to define extreme thermal conditions in summer and winter. As already mentioned, the 95th percentile was used for warm extremes and the 5th percentile for cool extremes both for Tair and thermal indices. The extreme warm thresholds were classified in levels 2 to 4 of indices’ assessment scales in summer and in levels − 3 to − 4 in winter (except for WCT that was 0).

**Table 3:** Air temperature and thermal indices’ thresholds for the definition of extreme thermal conditions in summer and winter. The thresholds are derived from hourly data recorded in 15 surface weather stations in the Athens metropolitan area, Greece

Index	Summer		Winter		Range of index levels
	Threshold (°C)	Category	Threshold (°C)	Category	
Tair	36.2	-	0.4	-	-
AT_warm_	37.7	2	-	-	0 to 4
HI	38	2	-	-	0 to 4
HU	42.6	3	-	-	0 to 5
NET	29.1	3	− 13.3	− 3	− 3 to 3
PET	48.5	4	− 7.9	− 4	− 4 to 4
UTCI	43.1	3	− 23.6	− 4	− 5 to 4
WBGT	33.1	4	-	-	0 to 4
WCT	-	-	− 4	− 1	− 6 to 0

Abbreviations:* AT*_*warm*_, apparent temperature; *HI*, heat index; *HU*, humidex; *NET*, net effective temperature; *PET*, physiologically equivalent temperature; *Tair*, air temperature; *UTCI*, universal thermal climate index; *WBGT*, wet-bulb globe temperature; *WCT*, wind chill temperature

**Fig. 4:**
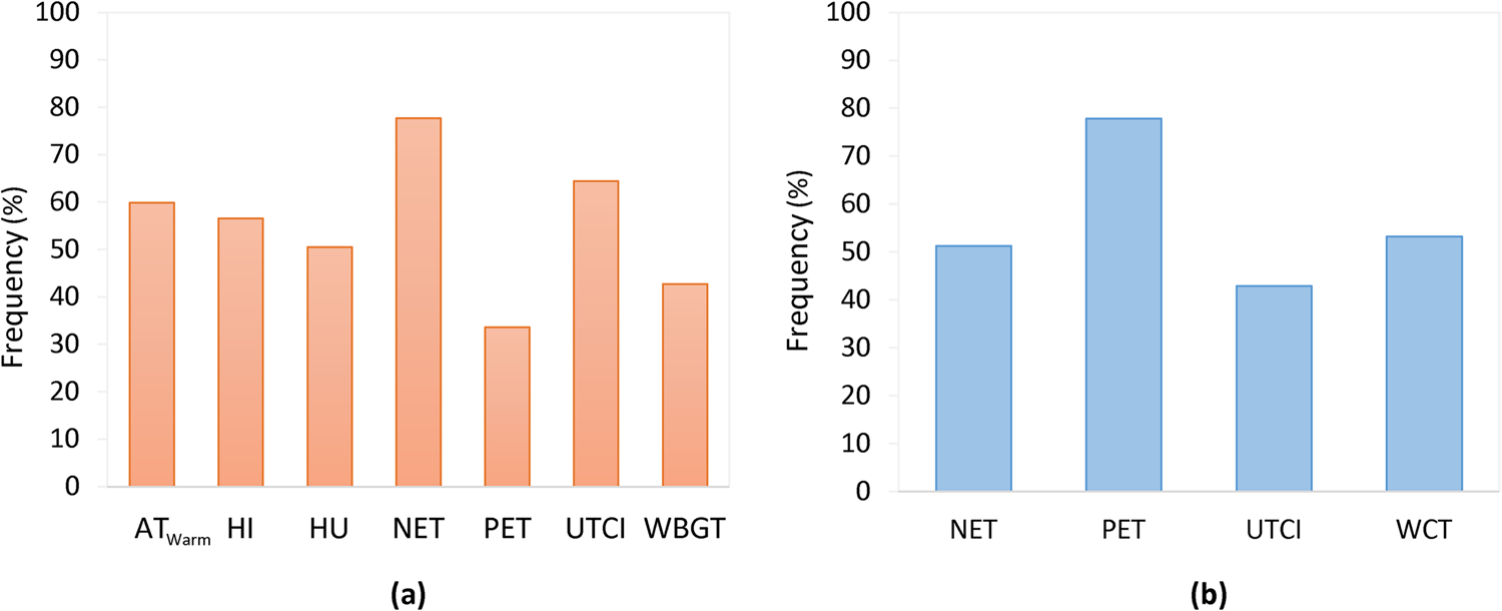
Occasions with extremely (**a**) warm conditions in summer and (**b**) cool conditions in winter identified jointly by thermal indices and air temperature

In total, 815 occasions were identified as extremely warm and 718 as extremely cool conditions according to Tair. Then, for the occurrences of warm/cool extremes in terms of Tair, we calculated the percentage of occasions that were also categorized as warm/cool extremes in terms of the thermal indices (Fig. 4). In extremely warm conditions in terms of Tair (Fig. 4a), NET and UTCI were also categorized as extreme 77.5% and 64.3% of the occasions, respectively. For PET, the agreement was found for 33.6% of the warm occasions. In the extreme cool conditions (Fig. 4b), NET and UTCI showed a lower agreement with Tair (51.3% and 42.9%, respectively) compared to warm extremes unlike PET which reached up to 77.9% of common occurrences with Tair.

## Discussion

The ability of a thermal index to assess the thermal environment has been a common concern among scientists for decades. Plenty of indices have been developed, and many studies have examined indices’ appropriateness for particular applications (Macpherson 1962; Gonzalez et al. 1974; Epstein and Moran 2006; Pantavou et al. 2013). However, there is no simple answer to address this issue. The aim, the type of assessment (i.e., sensation, discomfort, thermal stress, physiological effects), and the methodology of an application should be considered in order to select the appropriate one (Matzarakis 2021).

The present study examined the potential of eight popular thermal indices for the estimation of the thermal environment within a large Mediterranean city. The indices’ practicability and applicability were considered while the analysis focused on the sensitivity of indices commonly used for research and operational applications in the last decades. Emphasis was given to the potential of the indices to be routinely used so as, on a step further, to be incorporated in the operational forecasting process.

In terms of practicability, six out of the eight studied indices, namely, AT_warm_, HI, HU, NET, WBGT, and WCT, can be calculated by easily accessible meteorological variables. The rest two, PET and UTCI, require advanced estimations incorporating global solar radiation and calculation of radiation fluxes.

Variations in indices’ sensitivity were identified. AT_warm_ estimations missed to classify perception in the highest category of its assessment scale. WBGT attributed frequently thermal perception to high categories of its scale; however, WBGT effectiveness in reproducing extreme warm conditions in terms of Tair was relatively low. Thus, AT_warm_ and WBGT would be insufficient to capture differences in perception between extreme warm thermal conditions and heatwaves in the area of study providing often extreme estimations of thermal perception and potentially unrealistic alerts. WCT was found unsuitable for the prevailing weather condition of Athens mainly because of the restriction of estimations when only Tair < 10 °C but also because it is only applicable to cool conditions. HI and HU estimations in the two extreme categories were scarce. A shortcoming of HI and HU is the fact that they are applicable only to warm conditions, unlike NET, PET, and UTCI, which are applicable for both cool and warm conditions. PET showed a tendency of classifying thermal perception in the negative categories of the assessment scale due to possible increased humidity and wind sensitivity. PET values were classified relatively often in the cool categories and those with higher intensity of cold. Moreover, PET underperformed at estimating warm extreme conditions when Tair ranked thermal conditions as warm extremes.

According to the method followed, the indices studied, and the sample used, NET and UTCI satisfied requirements for operational use sufficiently. This is supported by the fact that NET:

- is estimated by a simple statistical formula involving commonly measured weather variables (i.e., Tair, Rh, WS),
- according to this study, it was sensitive in variations of both cool and warm conditions, showed similar sensitivity in variations of the thermal environment with the thermo-physiological indices PET and UTCI, and it was in good agreement with Tair for the estimation of warm extremes, and
- in a previous study in the Mediterranean climate, it showed good applicability for estimating the thermal perception reported by pedestrians in field surveys (Pantavou et al. 2013).

On the other hand, UTCI:

- is a thermo-physiological index,
- according to this study, it was found to be sensitive to the variations of the thermal environment, and in good agreement with Tair for the estimation of warm extremes,
- in a previous study, it showed the best applicability in the Mediterranean climate in estimating pedestrians’ thermal perception (Pantavou et al. 2013),
- has been recently incorporated in the operational forecasting systems at ECMWF and four European countries (Di Napoli et al. 2021).

The results of this analysis are in accordance with previous findings. In a comparative analysis of several thermal indices, UTCI was found to reproduce the variability of thermal conditions better than AT_warm_, HI, HU, NET, PET, WBGT, and WCT (Blazejczyk et al. 2012). Moreover, NET was found to correlate best with UTCI and to be more sensitive to the cooling effect of wind compared to UTCI (Blazejczyk et al. 2012). NET and UTCI were superior than PET in representing thermal perception of pedestrians in the Mediterranean climate (Pantavou et al. 2013). In a similar study in a humid subtropical climate, UTCI was also found better than PET in quantifying thermal response outdoors (Li et al. 2022) and determining the neutral temperature (Wei et al. 2022). Considering heat stress, WBGT showed substantially poorer effectiveness than PET and UTCI in health risk assessment for marathon runners (Thorsson et al. 2021), with PET showing the best applicability. In contrast to the present study, Zare et al. (2018) suggested a better correlation of UTCI with PET and WBGT, although NET was not included in the analysis. Moreover, AT and PET were identified as better predictors of heat- and cold-related mortality than UTCI (Morabito et al. 2014; Urban and Kyselý 2014).

A strength of the present study is that the analysis involved a long period comprising 12-year data. Moreover, it considered both warm and cool periods and included data from 15 surface weather stations within a large metropolitan area, comprising areas of variable microclimatic conditions (due to different physiographic conditions, proximity to the coast and urban characteristics) in order to expand the range of studied thermal conditions. The present study has some weaknesses. It was limited to the thermal environment of Athens, Greece, and tested the effectiveness of not all but some of the most widely used thermal indices. Not all weather stations directly measured solar radiation. To address this, we used data from the nearest station with a SR sensor. Additional indices such as PMV and SET* could be considered in the analysis, though this study focused mainly on indices found to be used operationally at the present in international climate and weather agencies.

## Conclusions

Thermal indices provide an integrated approach for assessing the thermal environment. They are valuable tools for quantifying the effect of the thermal environment on human and should be used on a regular basis in operational weather applications.

NET and UTCI have been found in the present study as effective for operational weather applications. In recent years, there is a trend towards the use of thermo-physiological indices and in particular of universal indices that could facilitate comparisons in different climate settings. The use of UTCI complies with this trend. UTCI is based on the multi-node “Fiala” thermoregulation model considering the behavioral adaptation of clothing insulation to ambient temperature. It is an international index, launched in 2009 in the framework of the International Society of Biometeorology to quantify the outdoor human perception for all climates. There is a wide range of applications already covered by UTCI with an insight for further development in the near future. On the other hand, NET is a simple index, easily calculated by Tair, RH and WS, probably less popular than UTCI in the use for operational weather applications but showing similar sensitivity in variations to thermal conditions with the thermo-physiological indices PET and UTCI.

The climate change and future scenarios suggest an increase in temperature and extreme thermal conditions. Thus, coordinated efforts should be made to enhance awareness and adaptation measures. Common and comprehensive tools for the assessment of how human body experiences atmospheric conditions such as thermal indices could greatly support this goal.

### Supplementary Information

Below is the link to the electronic supplementary material.

Supplementary file1 (DOCX 124 KB)

## Data Availability

The datasets used during the current study can be available from Dr K. Lagouvardos (email: lagouvar@noa.gr) on reasonable request.
