# Supplementary material for: Thermal environment and indices: an analysis for effectiveness in operational weather applications in a Mediterranean city (Athens, Greece)
Source: Int J Biometeorol. 2023 Nov 15;68(1):79–87. doi: 10.1007/s00484-023-02572-7 (PMC10752843; doi:10.1007/s00484-023-02572-7)
Supplement: Supplementary file 1 — Supplementary file1 (DOCX 124 KB) [file 484_2023_2572_MOESM1_ESM.docx]

**Supplementary Information**

**International Journal of Biometeorology**

**Thermal environment and indices: an analysis for effectiveness in operational weather applications** **in a Mediterranean city (Athens, Greece)**

Pantavou K.*, Kotroni V., Lagouvardos K.

Institute for Environmental Research and Sustainable Development, National Observatory of Athens, Penteli, 15236 Athens, Greece

*Corresponding author e-mail: kpantav@noa.gr

**Content**

**Table S1** Algebraic formulas used to calculate the thermal indices

**Table S2** Assessment scales of thermal indices suitable for cool or warm environmental conditions

**Table S3** Assessment scales of thermal indices suitable for both cool and warm environmental conditions

**Table S4** Characteristics of stations of the Automatic Weather Stations Network of the National Observatory of Athens in the Athens metropolitan area, Greece

**Figure S1** Mean relative humidity for each level of thermal indices’ assessment scale

**Figure S2** Mean wind speed for each level of thermal indices’ assessment scale

**Figure S3** Mean global solar radiation for each level of thermal indices’ assessment scale

**Table S1** Algebraic formulas used to calculate the thermal indices

| **Index (**°C**)** | **Formulas** |
| --- | --- |
| AT | Tair+0.33*VP-0.7*WS_10m_-4 |
| HI | -8.784695+1.61139411*Tair+2.338549*Rh-0.14611605*Tair*Rh-1.2308094*10^-2^*Tair^2^-1.6424828*10^-2^*Rh^2^+2.211732*10-^3^*Tair^2^*Rh+7.2546*10^-4^*Tair*Rh^2^-3.582*10^-6^*Tair^2^ *Rh^2^ |
| HU | Tair+(5/9)*(VP-10) |
| NET | 37-((37-Tair)/(0.68-0.0014*Rh+(1/(1.76+1.4*WS_1.2m_^0.75^))))-0.29*Tair*(1-0.01*Rh) |
| WBGT | 0.567*Tair+0.393*VP+3.94 |
| WCT | 13.12+0.6215*Tair-11.37*WS_10m_^0.16^+0.3965*Tair*WS_10m_*0.16 |

Source: Yaglou and Minard 1957; Masterson and Richardson 1979; Steadman 1984; Mayer and Höppe 1987; ASHRAE 1997; Li and Chan 2000; Fiala et al. 2001; BOM 2010; Bröde et al. 2012; NOAA 2017; Brimicombe et al. 2022

Abbreviations: AT, Apparent Temperature; HI, Heat Index; HU, Humidex; NET, Net Effective Temperature; Tair, air temperature (°C); Rh, relative humidity (%); VP: water vapor pressure (hPa); WBGT, Wet-Bulb Globe Temperature; WCI, Wind Chill Index; WS_1.2m_, Wind speed (m/s) at an elevation of 1.2 meters; WS_10m_: Wind speed (m/s) at an elevation of 10 meters

**Table S2** Assessment scales of thermal indices suitable for cool or warm environmental conditions

| **Warm conditions** | | | | | | | | | **Cool conditions** | | |
| --- | --- | --- | --- | --- | --- | --- | --- | --- | --- | --- | --- |
| **Index level** | **Apparent temperature (AT_Warm_)** | | **Heat Index (HI)** | | **Humidex (HU)** | | **Wet-Bulb Globe Temperature (WBGT)** | | **Index level** | **Wind Chill Temperature (WCT)** | |
|  | **Range (^o^C)** | **Category** | **Range (^o^C)** | **Category** | **Range (^o^C)** | **Degree of comfort** | **Range (^o^C)** | **Recommended Sporting Activity** |  | **Range (^o^C)** | **Exposure risk** |
| 0 | <27 | No danger | <27 | No danger | <30 | Little or no discomfort | < 18 | Unlimited | 0 | >0 | No risk |
| 1 | 27-32 | Fatigue possible with prolonged exposure and/or physical activity | 27–32 | Caution | 30-35 | Noticeable discomfort | 18 - 24 | Keep alert for possible increases in the index and for symptoms of heat stress | -1 | 0 to -9 | Low Risk |
| 2 | 32-39 | Heat stroke, heat cramps, or heat exhaustion possible with prolonged exposure and/or physical activity | 32–41 | Extreme caution | 36-40 | Evident discomfort | 23 - 28 | Active exercise for unacclimatised persons should be curtailed. | -2 | -10 to -27 | Moderate risk |
| 3 | 39-51 | Heat cramps or heat exhaustion likely, and heat stroke possible with prolonged exposure and/or physical activity | 41–54 | Danger | 41–45 | Great discomfort; avoid exertion | 28-30 | Active exercise for all but the well acclimatized should be curtailed. | -3 | -28 to -39 | High risk: exposed skin can freeze in 10 to 30 minutes |
| 4 | >51 | Heat stroke highly likely | ≥ 54 | Extreme danger | 46-54 | Dangerous; discomfort | > 30 | All training should be stopped | -4 | -40 to -47 | Very high risk: Exposed skin can freeze in 5 to 10 minutes |
| 5 |  |  |  |  | >54 | Heat stroke possible |  |  | -5 | -48 to -54 | Severe risk: exposed skin can freeze in 2 to 5 minutes |
|  |  |  |  |  |  |  |  |  | -6 | ≤-55 | Extreme risk: exposed skin can freeze in less than 2 minutes |

**Table** **S3** Assessment scales of thermal indices suitable for both cool and warm environmental conditions

| **Index level** | **Net Effective Temperature (NET)** | | **Physiologically Equivalent Temperature (PET)** | | | **Universal Climate Thermal Index (UTCI)** | |
| --- | --- | --- | --- | --- | --- | --- | --- |
|  | **Range (^o^C)** | **Category** | **Range (^o^C)** | **Thermal sensation** | **Grade of physiological stress** | **(^o^C)** | **Category** |
| -5 |  |  |  |  |  | <-40 | Extreme cold stress |
| -4 |  |  | <4 | Very cold | Extreme cold stress | -40 to -27 | Very strong cold stress |
| -3 | <1 | Very cold | 4-8 | Cold | Strong cold stress | -27 to -13 | Strong cold stress |
| -2 | 1-9 | Cold | 8-13 | Cool | Moderate cold stress | -13 to 0 | Moderate cold stress |
| -1 | 9-17 | Cool | 13-18 | Slightly cool | Slight cold stress | 0 to 9 | Slight cold stress |
| 0 | 17-21 | Comfortable | 18-23 | Neutral | No thermal stress | 9 to 26 | No thermal stress |
| 1 | 21-23 | Warm | 23-29 | Slightly warm | Slight heat stress | 26 to 32 | Moderate heat stress |
| 2 | 23-27 | Hot | 29-35 | Warm | Moderate heat stress | 32 to 38 | Strong heat stress |
| 3 | >27 | Very hot | 35-41 | Hot | Strong heat stress | 38 to 46 | Very strong heat stress |
| 4 |  |  | >41 | Very hot | Extreme heat stress | >46 | Extreme heat stress |

**Table S4** Characteristics of stations of the Automatic Weather Stations Network of the National Observatory of Athens in the Athens metropolitan area, Greece (Lagouvardos et al. 2017)

| **Stations** | **Latitude (degrees)** | **Longitude (degrees)** | **Altitude (m)** | **Start year** | **Global solar radiation** |
| --- | --- | --- | --- | --- | --- |
| Ampelokipoi | 37.981 | 23.758 | 136 | 2010 | ✓ |
| Athens | 37.978 | 23.715 | 50 | 2010 |  |
| Dionysos | 38.108 | 23.890 | 575 | 2010 |  |
| Faliro | 37.929 | 23.693 | 25 | 2012 |  |
| Kantza | 37.979 | 23.865 | 221 | 2010 |  |
| Lavrio | 37.711 | 24.055 | 2 | 2010 |  |
| Markopoulo | 37.888 | 23.928 | 104 | 2010 | ✓ |
| Maroussi | 38.048 | 23.810 | 235 | 2010 |  |
| Nea Makri | 38.058 | 23.976 | 90 | 2010 |  |
| Neos Kosmos | 37.959 | 23.732 | 85 | 2010 |  |
| Parnitha | 38.162 | 23.718 | 1230 | 2010 |  |
| Patissia | 38.021 | 23.729 | 90 | 2012 |  |
| Peristeri | 38.001 | 23.703 | 55 | 2012 |  |
| Psychico | 38.017 | 23.780 | 209 | 2010 |  |
| Spata | 37.982 | 23.909 | 120 | 2010 | ✓ |





**Figure S1** Mean relative humidity for each level of thermal indices’ assessment scale

**

**

**Figure S2** Mean wind speed for each level of thermal indices’ assessment scale





**Figure S3** Mean global solar radiation for each level of thermal indices’ assessment scale

**References**

ASHRAE (1997) American Society of Heating, Refrigerating and Air Conditioning Engineers Handbook Fundamentals

Brimicombe C, Di Napoli C, Quintino T, et al (2022) Thermofeel: A python thermal comfort indices library. SoftwareX 18:101005. https://doi.org/10.1016/j.softx.2022.101005

Bröde P, Fiala D, Błażejczyk K, et al (2012) Deriving the operational procedure for the Universal Thermal Climate Index (UTCI). Int J Biometeorol 56:481–494. https://doi.org/10.1007/s00484-011-0454-1

Bureau of Meteorology Australian Goverment (BOM) (2010) Thermal Comfort observations. http://www.bom.gov.au/info/thermal_stress/#apparent. Accessed 15 Mar 2023

Fiala D, Lomas KJ, Stohrer M (2001) Computer prediction of human thermoregulatory and temperature responses to a wide range of environmental conditions. Int J Biometeorol 45:143–159. https://doi.org/10.1007/s004840100099

Lagouvardos K, Kotroni V, Bezes A, et al (2017) The Automatic Weather Stations NOANN Network of the National Observatory of Athens: Operation and database. Geosci Data J 4:4–16. https://doi.org/10.1002/gdj3.44

Li PW, Chan ST (2000) Application of a weather stress index for alerting the public to stressful weather in Hong Kong. Meteorol Appl 7:369–375. https://doi.org/10.1017/S1350482700001602

Masterson J, Richardson F (1979) Humidex, a method of quantifying human discomfort due to excessive heat and humidity. Downsview, Ontario

Mayer H, Höppe P (1987) Thermal comfort of man in different urban environments. Theor Appl Climatol 38:43–49. https://doi.org/10.1007/BF00866252

NOAA (2017) Heat Index. http://www.nws.noaa.gov/os/heat/heat_index.shtml. Accessed 5 Jul 2017

Steadman RG (1984) A Universal Scale of Apparent Temperature. J Appl Meteorol Climatol 23:1974–1687. https://doi.org/10.1175/1520-0450(1984)023<1674:AUSOAT>2.0.CO;2

Yaglou CP, Minard D (1957) Control of heat casualties at military training centers. AMA Arch Intern Med 16:302–316
